# Inflammasome activation mediates inflammation and outcome in humans and mice with pneumococcal meningitis

**DOI:** 10.1186/1471-2334-13-358

**Published:** 2013-07-31

**Authors:** Madelijn Geldhoff, Barry B Mook-Kanamori, Matthijs C Brouwer, Dirk Troost, Jaklien C Leemans, Richard A Flavell, Arie Van der Ende, Tom Van der Poll, Diederik Van de Beek

**Affiliations:** 1 Department of Neurology, Center of Infection and Immunity Amsterdam (CINIMA), Academic Medical Center, University of Amsterdam, Amsterdam, The Netherlands; 2 Department of Neuropathology, Academic Medical Center, University of Amsterdam, Amsterdam, The Netherlands; 3 Department of Immunobiology, Yale University and Howard Hughes Medical Institute, New Haven, CT, USA; 4 Department of Medical Microbiology, Center of Infection and Immunity Amsterdam (CINIMA), Academic Medical Center, University of Amsterdam, Amsterdam, The Netherlands; 5 The Netherlands Reference Laboratory for Bacterial Meningitis, Center of Infection and Immunity Amsterdam (CINIMA), Academic Medical Center, University of Amsterdam, Amsterdam, The Netherlands; 6 Center for Experimental and Molecular Medicine (CEMM), Center of Infection and Immunity Amsterdam (CINIMA), Academic Medical Center, University of Amsterdam, Amsterdam, The Netherlands; 7 Division of Infectious Diseases, Center of Infection and Immunity Amsterdam (CINIMA), Amsterdam, University of Amsterdam, Amsterdam, The Netherlands

## Abstract

**Background:**

Inflammasomes are multi-protein intracellular signaling complexes that have recently been hypothesized to play a role in the regulation of the inflammation response. We studied associations between inflammasome-associated cytokines IL-1β and IL-18 in cerebrospinal fluid (CSF) of patients with bacterial meningitis and clinical outcome, and pneumococcal serotype. In a murine model of pneumococcal meningitis we examined the pathophysiological roles of two inflammasome proteins, NLRP3 (Nod-like receptor protein-3) and adaptor protein ASC (apoptosis-associated speck-like protein).

**Methods:**

In a nationwide prospective cohort study, CSF cytokine levels were measured and related to clinical outcome and pneumococcal serotype. In a murine model of pneumococcal meningitis using *Streptococcus pneumoniae* serotype 3, we examined bacterial titers, cytokine profiles and brain histology at 6 and 30 hours after inoculation in wild-type (WT), *Asc* and *Nlrp3* deficient mice.

**Results:**

In patients with bacterial meningitis, CSF levels of inflammasome associated cytokines IL-1β and IL-18 were related to complications, and unfavorable disease outcome. CSF levels of IL-1β were associated with pneumococcal serotype (p<0.001). In our animal model, *Asc* and *Nlrp3* deficient mice had decreased systemic inflammatory responses and bacterial outgrowth as compared to WT mice. Differences between *Asc*^−/−^ and WT mice appeared sooner after bacterial inoculation and were more widespread (lower pro-inflammatory cytokine levels in both blood and brain homogenate) than in *Nlrp3*^-/-^mice. *Nlrp3* deficiency was associated with an increase of cerebral neutrophil infiltration and cerebral hemorrhages when compared to WT controls.

**Conclusions:**

Our results implicate an important role for inflammasome proteins NLRP3 and ASC in the regulation of the systemic inflammatory response and the development of cerebral damage during pneumococcal meningitis, which may dependent on the pneumococcal serotype.

## Background

Bacterial meningitis is a life threatening infectious disease of the central nervous system that affects between 2.6 and 6.0 people per 100 000 per year in Europe and may be up to ten times higher in developing countries. The most common causative organism of community acquired bacterial meningitis in adults is *Streptococcus pneumoniae*, which is responsible for two-thirds of cases in Europe and United States [1]. Pneumococcal meningitis has a case fatality rate of 16%-37% and of the survivors 30-52% suffer from neurological sequelae [2,3]. There remains a need for better (adjuvant) therapies, for which further understanding of underlying pathophysiology is necessary [4].

Recently, several studies have examined the role of inflammasomes in bacterial meningitis. Inflammasomes are intracellular multiprotein complexes, belonging to the family of Nod-like receptors (NLRs) [5-7], and are triggered by exposure to microbial and endogenous danger signals such as ATP, changes in K^+^ concentration, oxygen radicals and uric acid released through cell injury in inflammation. Upon activation, NLRP3 binds to pro-caspase via adaptor apoptosis-associated speck-like protein (ASC), which is shared by several inflammasome types. Procaspase-1 is converted to activated caspase-1, which subsequently converts interleukins 1beta (IL-1β) and IL-18 into their active secreted forms [5-7]. Recently however, caspase-independent proinflammatory activity of NLRP3 has also been described [8,9]. To date, four inflammasomes have been characterized, of which NLRP3 has been the most extensively researched.

Further examination of the role of inflammasomes in pneumococcal meningitis is of interest for several reasons: First, inflammasomes are the well established activators of caspase-1, which has been shown to be elevated in the cerebral spinal fluid (CSF) of patients with pneumococcal meningitis compared to non-infected controls [10,11]. Moreover, mice deficient of caspase-1 displayed less severe inflammation, decreased brain water content and improved clinical score in a pneumococcal meningitis model [10,12]. Second, IL-1β, which is activated by caspase-1, has been shown to be elevated in the CSF of children with pneumococcal meningitis and correlates with disease severity [11], a finding that also has been observed in various animal models [13-15]. Lastly, several murine models have demonstrated the importance of NLRP3 in the pathophysiology of invasive pneumococcal disease [16,17]. Most notably, a recent study showed that NLRP3 mediates brain damage in an experimental meningitis model using a serotype 2 *S*. *pneumoniae*[18].

In this study we measured the CSF levels of inflammasome related cytokines IL-1β and IL-18 in a prospective nationwide cohort of community acquired bacterial meningitis and correlated these to clinical data and pneumococcal serotype. We then investigated the role of inflammasome gene NLRP3 and adapter protein ASC in a murine model of meningitis using serotype 3 *S*. *pneumoniae*, a common serotype in pneumococcal meningitis [19].

## Methods

### Patients cohort

In a nationwide prospective cohort study we included bacterial meningitis patients older than 16 years of age with positive CSF cultures who were identified by The Netherlands Reference Laboratory for Bacterial Meningitis (NRLBM) from March 2006 to June 2009. The NRLBM provided the names of the hospitals where patients with bacterial meningitis had been admitted 2–6 days previously. The treating physician was contacted, and informed consent was obtained from all participating patients or their legally authorized representatives. Outcome was graded at discharge according to the Glasgow Outcome Scale, a well-validated instrument with good interobserver agreement [20]. A score of one on this scale indicates death; a score of two a vegetative state; a score of three severe disability; a score of four moderate disability; and a score of five mild or no disability. A favorable outcome was defined as a score of five, and unfavorable outcome as a score of one to four. The study was approved by the medical ethical (review) committee of the Academic Medical Center of Amsterdam.

### IL-1β and IL-18 measurements in CSF samples of patients with bacterial meningitis

We measured IL-1β and IL-18 in the CSF of 289 patients with bacterial meningitis included in the cohort and 19 controls with luminex® technology using a Milliplex assay (Millipore, Billerica, MA, USA). CSF from the first diagnostic tap was collected, centrifuged and supernatant was aliquoted and stored at −80°C until analysis. Controls were patients evaluated for acute headache, without signs of meningitis and normal CSF findings. In these patients a subarachnoid hemorrhage was excluded as cause of their headache by CSF examination. Leftover CSF was collected, centrifuged and supernatant was stored at −80°C until analysis.

### Mouse model and tissue preparation

A well characterized and previously described murine model of pneumococcal meningitis was used in this study [21]. *Nlrp3*^−/−^ mice with C57BL/6 background (kind gift of Richard Flavell, Howard Hughes Medical Institute, Yale University School of Medicine, New Haven, CT, USA) and *Asc*^−/−^ mice with C57BL/6 background (kind gift of Fayyaz Sutterwala, University of Iowa, Iowa City, IA, USA), and specific pathogen-free C57BL/6 mice (Charles River, Wilmington, MN, USA) were weighed, clinically examined, and scored clinically. Inoculations were performed in several rounds, all with male mice aged 8–12 weeks. In each inoculation round knockout mice and an equal number of wild-type mice were inoculated with the same bacterial inoculum to control for variations between inocula. Experiments were approved by the Institutional Animal Care and Use Committee of the Academic Medical Center, Amsterdam, The Netherlands.

Serotype 3 *S*. *pneumoniae* (ATCC 6303; American Type Culture Collection, Rockville, MD, USA) was grown to mid log phase in 4 hours at 37°C in Todd-Hewitt broth supplemented with yeast (Difco, Detroit, MI). Pneumococci were harvested by centrifugation at 4000 rpm for 10 min, and washed twice with sterile isotonic saline. Bacteria were diluted to a final concentration of 1x10^6^ CFU/ml and serial ten-fold dilutions were plated on sheep blood agar plates for quantification.

Mice were inoculated in the cisterna magna under isoflurane anesthesia with 10 μl saline containing 1×10^4^ CFU (range 0.6×10^4^ – 1.2×10^4^ CFU) of *S*. *pneumoniae* or sterile saline alone. Twelve mice per group (WT, *Nlrp3*^−/−^ and *Asc*^−/−^) were inoculated with *S*. *pneumoniae* and six mice per group (WT, *Nlrp3*^−/−^ and *Asc*^−/−^) with sterile saline. After intracisternal inoculation mice were assessed for neurologic damage as a result of the puncture, which was not present in any of the mice. At 6 or 30 hours post infection mice were anesthetized by intraperitoneal injection of ketamine (Eurovet Animal Health, Bladel, the Netherlands) and medetomidine (Pfizer Animal Health, Capelle aan den Ijssel, the Netherlands) followed by cardiac puncture for blood collection and perfusion of organs with sterile isotonic saline via the left ventricle.

CSF was collected by puncture of the cisterna magna, and brains, lungs and spleen were harvested. The right hemisphere was suspended in 10% buffered formalin and embedded in paraffin for histopathology. The left hemisphere and spleen were taken up in 20% weight per volume sterile saline and were disrupted with a tissue homogenizer. Serial ten-fold dilutions of blood, CSF, brain homogenate and spleen homogenate were plated on sheep-blood agar plates and bacteria were allowed to grow overnight at 37°C. Heparin blood was centrifuged at 4000 rpm for 5 min. at 4°C. Tissue homogenates were lysed by adding 1:1 two times concentrated lysis buffer (150 mM NaCl, 15 mM Tris, 1 mM MgCl(H_2_O)_6_, 1 mM CaCl_2_(H_2_O)_2_, 1% Triton, AEBSF 4 μg/ml, EDTA-NA2 50 μg/ml, pepstatin 10 ng/ml, leupeptin 10 ng/ml, pH 7.4), incubating on ice for 30 min. and centrifuged at 4000 rpm for 5 min. at 4°C. Plasma and lysed tissue supernatant were removed and stored at −20°C for further analysis.

### RT-PCR

Total RNA was isolated from murine brain homogenates with the Nucleospin® RNA II Purification kit (Clontech Laboratories, Mountain View, CA, USA; Bioke, Leiden, the Netherlands). Isolated RNA was converted to cDNA using oligo(dT) primer (Promega, Leiden, the Netherlands), Moloney murine leukemia virus reverse transcriptase (Invitrogen, Breda, the Netherlands), RT-buffer (Promega, Leiden, the Netherlands), deoxynucleotide triphosphate mix (Invitrogen, Breda, the Netherlands), dithiothreitol (Duchefa Farma, Haarlem, the Netherlands) and RNAse inhibitor (Invitrogen, Breda, the Netherlands). After incubation for 10 min at 23°C, RT was carried out for 60 min at 42°C, followed by RT inactivation for 3 min at 95°C. Reverse transcription-PCR’s (RT-PCRs) were performed with LightCycler SYBR green I master mix (Roche, Mijdrecht, the Netherlands) and measured in a LightCycler 480 (Roche) apparatus under the following conditions: 5 min 95°C hot start, followed by 40 cycles of amplification (95°C for 15 sec - 60°C for 5 sec - 72°C for 20 sec). For quantification, standard curves were constructed on serial dilutions of a sample with known high cDNA content. Data were analyzed using LightCycler software. Gene expression is presented as a ratio of the expression of the housekeeping gene Glyceraldehyde-3-phosphate dehydrogenase (GAPDH). The primers used for RT-PCR were as follows: mouse GAPDH, 5'-CTCATGACCACAGTCCATGC-3' (forw) and 5'-CACATTGGGGGTAGGAACAC-3' (rev); mouse Nlrp3, 5'-CCACAGTGTAACTTGCAGAAGC-3' (forw) and 5'- GGTGTGTGAAGTTCTGGTTGG-3' (rev); mouse Asc, 5'-GGTGTGTGAAGTTCTGGTTGG-3' (forw) and 5'-GGTGTGTGAAGTTCTGGTTGG-3' (rev).

### Cytokine measurements in murine tissue

IL-1β, IL-6, KC, TNF-α, IL-18, and MIP-2 were measured in plasma and brain homogenates with luminex® technology using a mouse cytokine and chemokine Bioplex kit (Bio-Rad Laboratories, Veenendaal, The Netherlands). Luminex assays were analysed on a Luminex 200 with Bio-Plex Manager software 5.0. Samples were 4 times diluted. Mouse myeloperoxidase (MPO) was measured by ELISA (Hycult Biotechnology, Uden, The Netherlands). Mouse albumin was measured by ELISA (GenWay Biotech, San Diego, CA).

### Murine histopathology

Five μm paraffin brain sections were cut in a coronal plane from the olfactory bulb to the beginning of the cerebellum, and sections at intervals of 1400 μm or intervals of 700 μm throughout the hippocampal region were selected. Sections were mounted on slides and stained with hematoxylin and eosin. To assess differences in brain damage, coronal cut brain sections of WT and *Asc*^−/−^ and *Nlrp3*^−/−^ mice were scored for intracerebral hemorrhages, subpial hemorrhages, cerebral infarctions, and for neutrophil influx on a five point scale: 0) normal histopathology; 1) few inflammatory cells in the meninges but no perivascular cuffing; 2) moderate number of inflammatory cells in the meningitis and cuffing of some of the vessels; 3) extensive number of inflammatory cells in the meninges, prominent perivascular cuffs with mild infiltration of the neutrophil; 4) extensive number of inflammatory cells in the meninges, prominent perivascular cuffs, the presence of many inflammatory cells in the neutrophil and intraparenchymal pocket formation. Sections of mice 30 hours after induction of pneumococcal meningitis (n=8 per group) were scored. Sections were scored by two independent observers blinded for the experimental groups (interobserver kappa 0.75).

### Statistics

The Mann–Whitney *U* test was used to identify differences in baseline characteristics, bacterial outgrowth, cytokine levels and histopathological scores among groups with respect to continuous variables. Dichotomous variables were compared using the *χ*2 test. Correlation analyses were performed with the Spearman’s rank correlation coefficient. For all analyses a P-value < 0.05 was considered significant.

## Results

### CSF IL-1β and IL-18 levels in patients with bacterial meningitis are associated with complications and unfavorable disease outcome

A total of 801 Dutch patients with bacterial meningitis were included as described previously [22]. In this study the distribution of causative organisms was: 576 episodes (72%) *S*. *pneumoniae*, 92 (12%) *Neisseria meningitidis*, 41 (5%), *Listeria monocytogenes*, and other bacteria in 92 (12%) episodes. The case fatality rate was 18%, and 38% of patients had poor clinical functional outcome as defined as a score of 1–4 on the Glasgow Outcome Scale. CSF was available in 289 of the episodes with bacterial meningitis (36%), and 211 of 576 with pneumococcal meningitis (35%). Levels of IL-1β and IL-18 were elevated in the CSF of patients with meningitis as compared to controls. Higher IL-1β levels were associated with occurrence of systemic complications (median 0.91 ng/ml [IQR 0.15-3.00] versus 2.02 ng/ml [IQR 0.33-5.26], p=0.001) and neurologic complications (median 0.81 ng/ml [IQR 0.15-3.36] versus 1.60 ng/ml [IQR 0.29-4.73], p=0.020). IL-1β levels were higher in patients with an unfavorable outcome although this difference was not statistically significant (median 1.04 ng/ml [IQR 0.17-3.65] versus 1.53 ng/ml [IQR 0.27-5.16], p=0.11). High IL-18 levels were also associated with systemic complications (median 8.50 ng/ml [IQR 3.07-20.71] versus 15.13 ng/ml [IQR 6.35-27.32], p=0.006) and unfavorable outcome (median 9.27 ng/ml [IQR 3.36-22.83] versus 14.65 ng/ml [IQR 5.53-26.97], p=0.037). In the subgroup of patients with pneumococcal meningitis (n=211) associations with systemic complications remained significant.

### CSF IL-1β and IL-18 levels in patients with bacterial meningitis are associated with pneumococcal serotype

**Figure 1:**
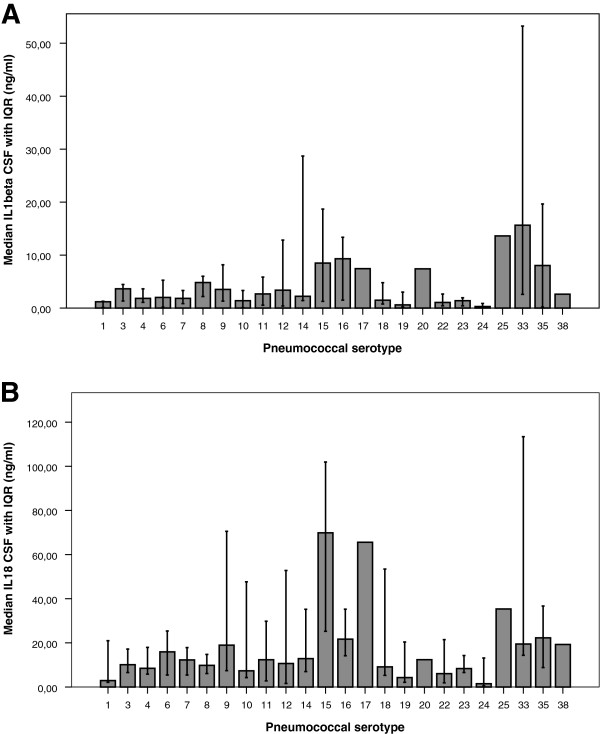
**Median levels of IL-1β (A) and IL-18 (B) with interquartile range by pneumococcal serotype.** CSF levels of IL-1β and IL18 were related to pneumococcal serotype (Kruskal-Wallis 1 way ANOVA, p<0.001).

Pneumococcal serotyping was performed in 509 pneumococcal strains (88%) and the most common serotypes were 3, 23 and 7 (Table III; serotype distribution has been partly published previously [23]). CSF levels of IL-1β were related to pneumococcal serotype (Kruskal-Wallis 1 way ANOVA, p<0.001; Figure 1).

### ASC and NLRP3 expression in Asc and Nlrp3 knockout mice

**Figure 2:**
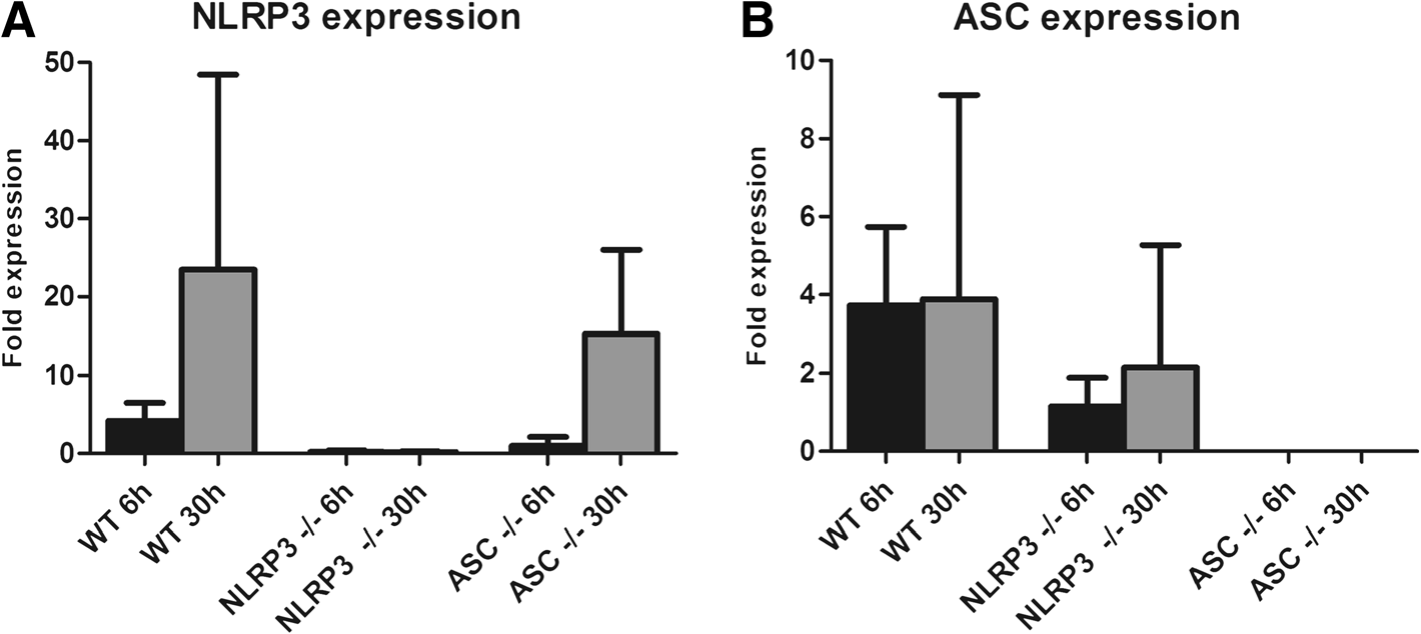
**Expression of NLRP3 and ASC in brain homogenates.** Expression of NLRP3/GAPDH **(A) **and ASC/GAPDH **(B)** in brain homogenates of WT, *Asc*^−/−^ and *Nlrp3*^−/−^ mice 6 hours and 30 hours after induction of *S*. *pneumoniae* meningitis compared to mice inoculated with saline. Three mice per group were analysed in the sham infected mice and the 6 hour timepoint; 4 mice were analyzed at the 30 hour timepoint. Data are given as means +/− SD.

To confirm that the inflammasome components ASC and NLRP3 were expressed or knocked out in our meningitis mouse model, we examined mouse brain homogenates from WT mice infected with *S*. *pneumoniae* serotype 3. At 6 and 30 h after infection ASC and NLRP3 expression in brain homogenates appeared upregulated as compared to saline inoculated mice (Figure 2). *Nlrp3*^−/−^ mice showed expression of ASC and no NLRP3, and conversely *Asc*^−/−^ mice showed expression of NLRP3 and no ASC.

### Decreased systemic bacterial loads in Asc and Nlrp3 knockout mice

**Figure 3:**
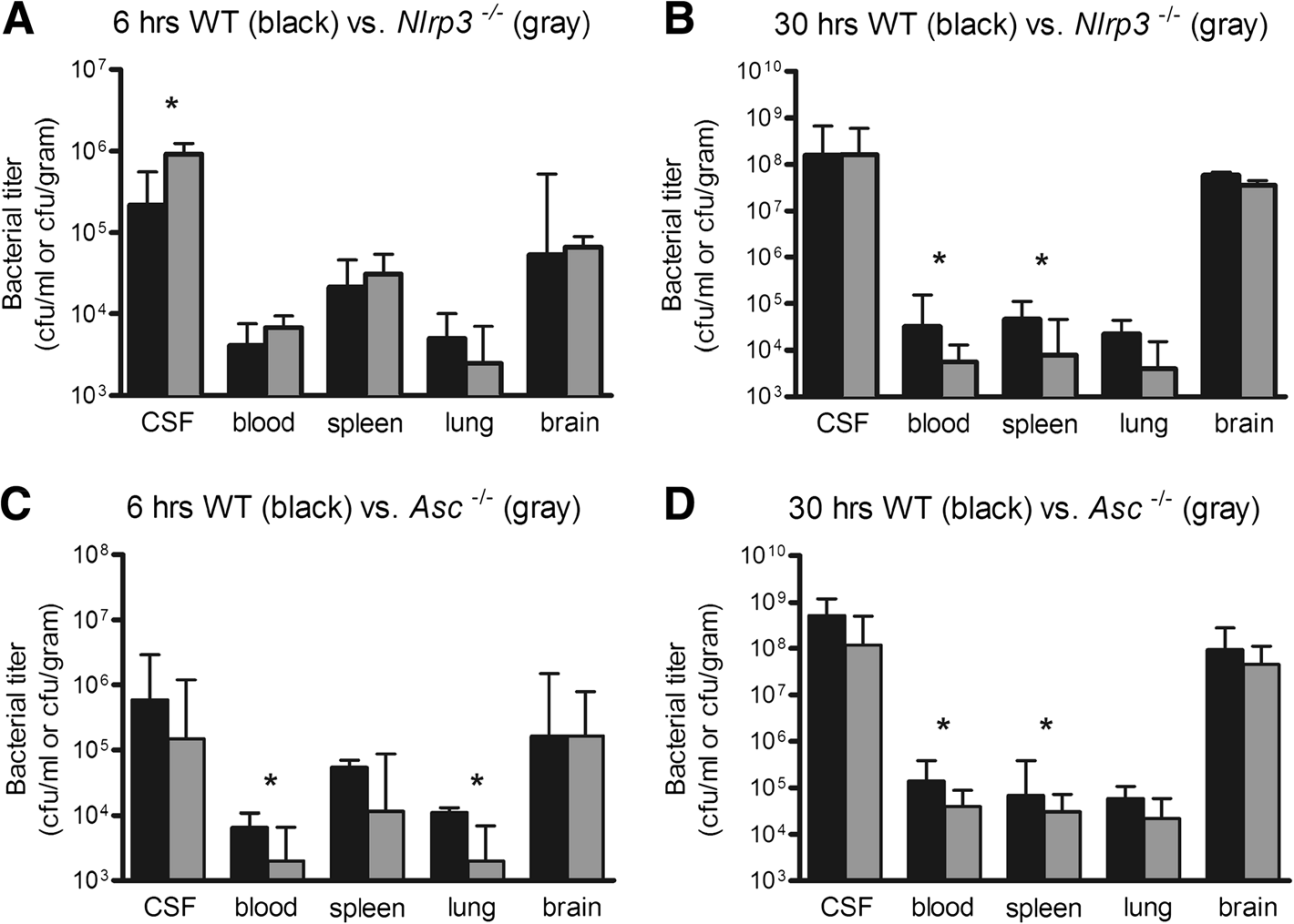
**Bacterial outgrowth in blood, spleen, lung brain and CSF.** Bacterial titers in WT vs., Nlrp3^−/−^ mice at 6 **(A)** and 30 hours **(B)**; and WT vs. and Asc^−/−^ mice at 6 **(C)** and 30 hours **(D)** after induction of pneumococcal meningitis. Twelve mice per group were analyzed. Data are given as medians and 75th quartile. * P < 0.05.

*Nlrp3*^−/−^ mice showed more bacterial outgrowth in CSF at 6 h compared to WT mice (median 9.2 x 10^5^ CFU/ml versus 2.2 x 10^5^ CFU/ml, p=0.046 Figure 3A). However, *Nlrp3*^−/−^ had less bacterial outgrowth in blood and spleen at 30 h, as compared to WT mice (blood, 5.7 × 10^3^ CFU/ml versus 3.2 × 10^4^ CFU/ml, p=0.017; spleen, 8.0 × 10^3^ CFU/ml versus 4.7 × 10^5^ CFU/ml, p=0.012 Figure 3B). No differences in bacterial outgrowth in brain homogenates were observed. *Asc*^−/−^ mice showed less bacterial outgrowth at 6 h in blood and lung compared to WT mice (blood, 2.0 × 10^3^ CFU/ml versus 6.6 × 10^3^ CFU/ml, p=0.017; lung, 2.0 × 10^3^ CFU/ml versus 1.1 × 10^5^ CFU/ml, p=0.043 Figure 3C). At 30 h *Asc*^−/−^ mice showed less bacterial outgrowth in blood (3.9 x 10^4^ CFU/ml versus 1.4 × 10^5^ CFU/ml, p=0.039 Figure 3D) and spleen (3.1 × 10^4^ CFU/ml versus 6.7 × 10^4^ CFU/ml, p=0.016). No differences in bacterial outgrowth in CSF or brain homogenates in *Asc*^−/−^ mice were observed.

### Decreased systemic inflammatory response in both Nlrp3 and Asc knockout mice at 6 and 30 hrs

**Figure 4:**
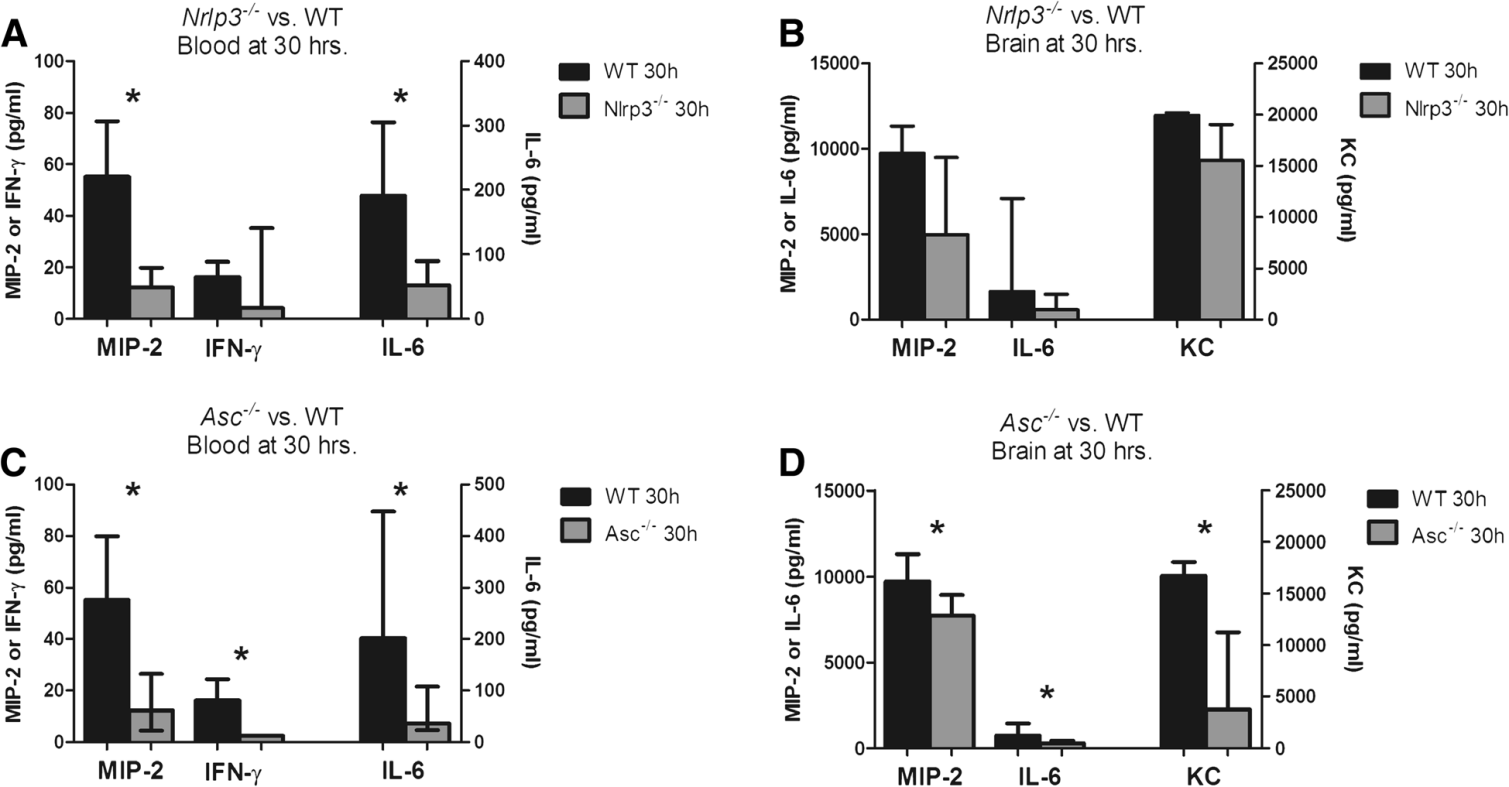
**Cytokine levels in blood and brain homogenate.** Cytokines were measured in *WT* (*n* = *11*), *Nlrp3*^−/−^ (n = 12) and *Asc*^−/−^ (n = 12) mice in blood **(A**, **C)** and brain homogenate (*WT*; *n* = *7*, *Nlrp3*^−/−^; n = 8, *Asc*^−/−^; n = 8) **(B**, **D)** at 30 hours after induction of pneumococcal meningitis. Undisplayed cytokines measurement did not differ significantly between *Nlrp3*^−/−^ or *Asc*^−/−^ and WT mice. Data are given as medians and 75th quartile. * P < 0.05.

*Nlrp3*^−/−^ mice showed decreased plasma levels of MIP-2 (median 12 pg/ml [IQR 5–20] versus 55 pg/ml [IQR 5–77], p=0.037) and IL-6 (52 pg/ml [IQR 24–90] versus 191 pg/ml [70–306], p=0.019) at 30 h as compared to WT mice (Figure 4A). No significant cytokine differences were found at 6 hours. *Asc*^−/−^ mice showed decreased plasma levels of MIP-2 (12 pg/ml [IQR 5–27] versus 55 pg/ml [IQR 35–80] pg/ml, p=0.01), IL-6 (37 pg/ml [IQR 24–108] versus 202 pg/ml [IQR 46–448], p=0.034) and IFN- γ (3 pg/ml [3] versus 16 pg/ml [3-24], p=0.005) at 30 h, as compared to WT mice. At 6 h only KC levels were lower (105 pg/ml [78–202] versus 186 pg/ml [151–388], p=0.005). Notably, plasma levels of IL-1β and IL-18 were similar in *Nlrp3*^−/−^, *Asc*^−/−^ and WT mice (Figure 4C). For the other measured cytokines no significant difference was found at 6 or 30 hours.

### Decreased brain cytokine and chemokine levels in Asc^−/−^ but not Nlrp3^−/−^ mice

*Nlrp3*^−/−^ mice showed no differences in cytokine responses in the brain compared to WT mice, and brain albumin levels were also similar between WT and *Nlrp3*^−/−^ mice (data not shown). *Asc*^−/−^ mice displayed lower levels of KC (median, 3.81 ng/ml [IQR 1.57-11.24] versus 16.75 ng/ml [IQR 11.49-18.12], p=0.040), MIP-2 (7.74 ng/ml [IQR 1.46-8.91] vs. 9.74 ng/ml [IQR 8.20-11.3], p=0.049) and IL-6 (0.30 ng/ml [IQR 0.10-0.45] versus 0.76 ng/ml [IQR 0.61-1.45], p=0.049) than WT mice in brain homogenates at 30 h (Figure 4D). Consistent with this finding, brain albumin concentrations were decreased in *Asc*^−/−^ mice compared to WT mice at 30 h (p=0.026), indicating attenuated blood brain barrier disruption in *Asc*^−/−^ mice compared to WT mice. No differences in brain IL-1β or IL-18 levels were measured between WT and *Nlrp3*^−/−^ or *Asc*^−/−^ mice.

### Enhanced brain damage in Nlrp3^−/−^ but not in Asc^−/−^ mice

**Figure 5:**
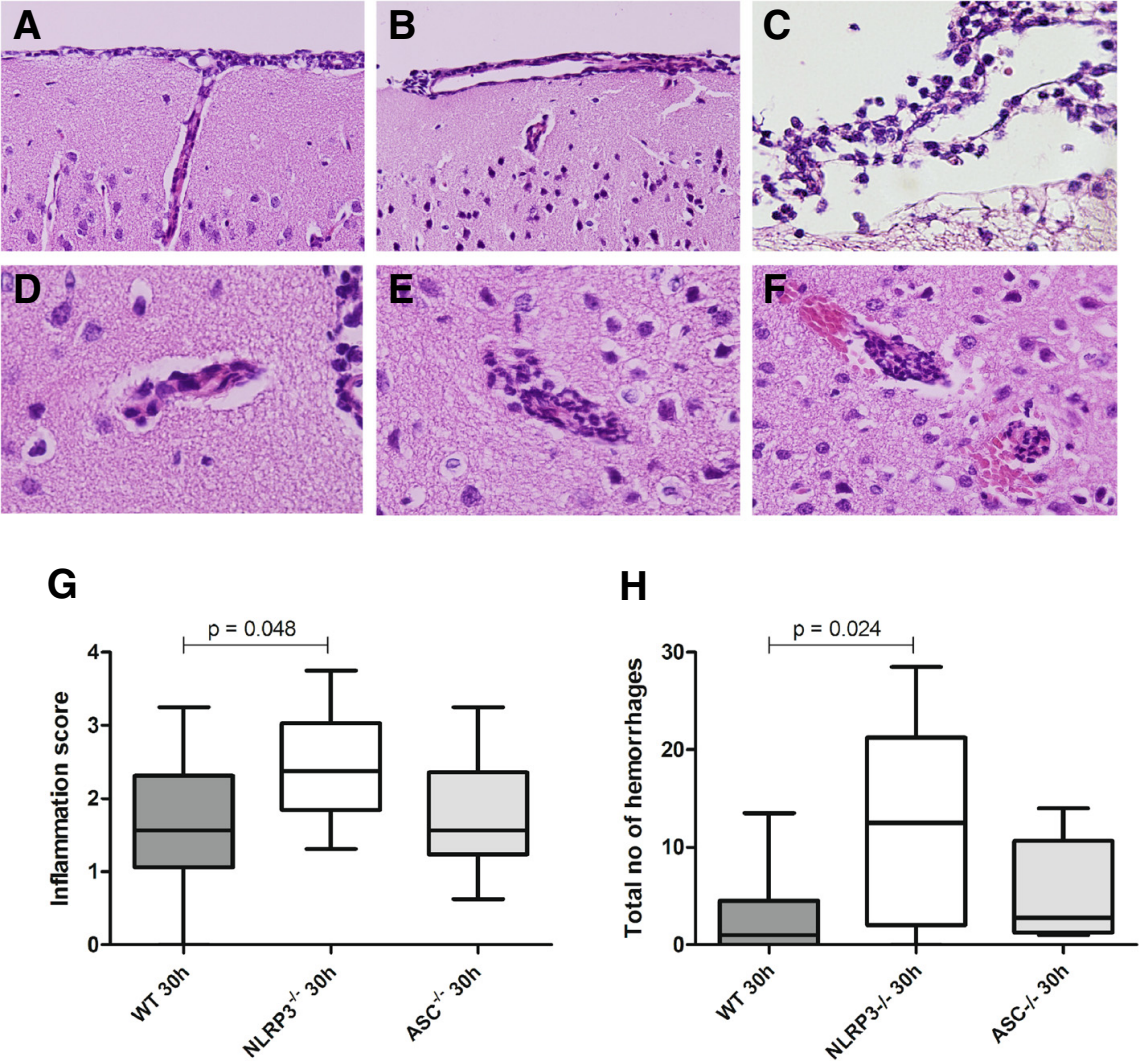
**Histopathology in *Asc***^−/−^**, *Nlrp3***^−/−^**, and WT mice with pneumococcal meningitis.** Representative brain slides showing neutrophil infiltration in the meninges 30 hours after induction of pneumococcal meningitis in a WT mouse **(A)**, *Asc*^−/−^ mouse **(B)** and *Nlrp3*^−/−^ mouse **(C)**. Perivascular cuffing 30 hours after induction of pneumococcal meningitis in a WT mouse **(D)**, *Asc*^−/−^ mouse **(E)** and *Nlrp3*^−/−^ mouse** (F)**, showing frequent intracerebral and subpial hemorrhages associated with neutrophil infiltration; meningeal, perivascular and intracerebral neutrophil influx was scored on a scale 0–4, means of 16 brain slides per mouse in the coronal plane are displayed **(**WT, n = 11; *Asc*^−/−^, n = 8; *Nlrp3*^−/−^, n = 9; **G)**. Sum of intracerebral hemorrhages and subpial hemorrhages per mouse **(**WT, n = 11; *Asc*^−/−^; n = 8; *Nlrp3*^−/−^; n = 9; **H)**.

Neutrophil infiltrate in the brain was more pronounced in *Nlrp3*^−/−^ mice at 30 h after inoculation as compared to WT mice (median score 1.5 versus 2.4, p=0.018; Figure 5G). *Nlrp3*^−/−^ mice also showed an elevated number of intracerebral and subpial hemorrhages and as compared to WT mice (median 12.5 versus 1.0 per slide, p=0.02; Figure 5H). *Asc*^−/−^ mice showed no difference in neutrophil influx score and intracerebral hemorrhages compared to WT mice. Brain MPO levels were similar in both knockouts and wild-type mice (data not shown).

## Discussion

This study implicates an important role for inflammasomes in regulation of systemic inflammation and development of cerebral damage during pneumococcal meningitis. In our patient cohort, inflammasome associated cytokines IL-1β and IL-18 levels in CSF of patients with bacterial meningitis correlated with development of systemic complications and unfavorable prognosis; and in the subgroup of patients with pneumococcal meningitis, IL-1β and IL-18 correlated with systemic complications only. In our murine model of pneumococcal meningitis, deficiency of inflammasome components ASC and NLRP3 led to decreased systemic inflammatory responses and bacterial outgrowth in the systemic compartment as compared with WT mice. Conversely, *Nlrp3* deficiency led to enhanced central nervous system inflammation and increased brain damage. Differences between *Asc*^−/−^ and WT mice occurred sooner after intrathecal inoculation with *S*. *pneumoniae* (lower bacterial titers and KC serum levels at 6 h) and were more widespread (lower pro-inflammatory cytokine levels in both the systemic compartment (blood) and central nervous system compartment (brain homogenate)) than in the *Nlrp3*^-/-^mice.

In our murine model, NLRP3 was protective for brain damage, as *Nlrp3*^−/−^ mice had enhanced cerebral neutrophil influx and an increased number of cerebral hemorrhages. NLRP3 has been investigated with regard to pneumococcal infections in both lung infection models and a meningitis model [17,18,24], but findings are not unanimous. In a lung-infection model, *Nlrp3*^−/−^ mice have higher bacterial titers and a higher mortality than WT controls. In a murine model of pneumococcal meningitis, better clinical outcome and decreased brain inflammation in *Nlrp3*^−/−^ (and *Asc*^−/−^) mice was found as compared to WT controls [18]. Blocking of IL-1β and IL-18 in this meningitis model, led to a decrease in disease severity and which prompted the suggestion that the NLRP and ASC dependent changes are solely IL-1 and IL-18 related [18]. Our findings that IL-1β and IL-18 levels were not significantly altered in *Nlrp3*^−/−^ or *Asc*^−/−^ mice, must be interpreted with caution as no assays are available that can discriminate between the pro- and active forms of murine IL-1β and IL-18. Previous studies showed increased IL-1β levels in brain homogenates of WT mice with pneumococcal meningitis at 30 hours compared to sham [21]. We did not perform experiments blocking IL-1β, IL-18 or Caspase-1 in our model to further elucidate this mechanism.

Discrepancies in brain damage in *Nlrp3*^−/−^ mice between our study and the previous study in experimental pneumococcal meningitis may be explained by the different pneumococcal serotypes used to establish meningitis between both models [18]. We inoculated mice with a *S*. *pneumoniae* serotype 3 strain as opposed to the serotype 2 strain used in the previous study. Furthermore, we used a lower intrathecal dose of *S*. *pneumoniae* than the previous study (10^4^ CFU vs. 10^5^/10^6^ CFU). *S*. *pneumoniae* serotype 2 is less heavily encapsulated and less virulent than serotype 3 and needs high doses to induce infection [25]. An *in vitro* study showed that high bacterial loads of *S*. *pneumoniae* serotype 2 are needed before IL-1β concentration in cell culture supernatants are elevated [26].

The variation of immune response between different serotypes of *S*. *pneumoniae* has been demonstrated by several groups [25,27]. In our patient cohort, we observed that the most common pneumococcal serotypes were 3, 23, and 7, and that CSF levels of IL-1β was serotype related. This observation may be due to serotype specific properties of the pneumococcal capsule. Alternatively, pneumolysin, a pore-forming toxin which is known to interact directly with the innate immune system (through, for instance complement or binding of Toll Like Receptor-4), is secreted in varying amounts depending on bacterial serotype. Pneumolysin has been reported to have both inflammasome inhibiting and activating properties [16,17,28], which may be caused by the recently described effects of pneumolysin polymorphisms on innate immune system recognition [17]. We chose *S*. *pneumoniae* serotype 3 for our animal studies, as it is one of the most commonly encountered serotypes among patients with pneumococcal meningitis [19].

The more pronounced phenotype of the *Asc*^−/−^ mice as compared to the *Nlrp3*^−/−^ mice with pneumococcal meningitis can be explained by other, NLRP3 inflammasome independent, functions of ASC. The (functional) relationship between ASC, NLRP3, and caspase-1 activation during pneumococcal infection was recently described in murine pneumonia model [24], in which *S*. *pneumoniae* infection led to caspase-1 activation and IL-1β/IL-18 maturation through the activation of both the NLRP3 *and* the AIM2 (absent in melanoma) inflammasomes, in a process which was completely absent in the ASC deficient mice. Furthermore, ASC is capable of binding and facilitating the function of several other inflammasomes (such as NLRC4 and IFI16), though the relevance of this during pneumococcal infection is not evident [29]. Lastly, independently of the inflammasomes, ASC has been shown to potent regulator of a large number of inflammatory and cell-death related genes [30]. The observation that NLRP3 deficient mice but not ASC deficient mice, expressed more brain damage suggests a protective mechanism in which NLRP3 may act independently of ASC and of the NLRP3 inflammasome. Such “inflammasome-independent” role of NLRP3 in tissue injury has been described in a mouse model of renal ischemia-reperfusion injury [8,9], though a mechanism remains unclear.

## Conclusion

In conclusion, although a definite mechanism remains elusive, our results provide additional evidence for an important role of inflammasomes (specifically the NLRP3 and ASC proteins and inflammasome associated cytokines IL-1β and IL-18) in the regulation of an inflammatory response and brain damage during pneumococcal meningitis. Further human and animal studies are necessary to clarify the pathophysiological mechanism, as well as explore the possibility of interference of inflammasome activation as a potential adjunctive therapy in the treatment of pneumococcal meningitis.

## Competing interests

The authors have no conflicts of interest.

## Authors’ contributions

MG and BM-K participated equally in the planning and conducting of all the herein mentioned experiments, as well as the writing of the manuscript. MB aided in the data analysis as well as drafting of this manuscript. DT and JL aided in the histological analyses. RF provided the transgenic mice needed for the murine studies. AE aided in the processing and analysis of cerebral spinal fluid. TP and DB conceived of the study, participated in design and execution and evaluation of the various experiments. DB provided funding and aided in the drafting of this manuscript. All authors read and approved the final manuscript.

## Pre-publication history

The pre-publication history for this paper can be accessed here:

http://www.biomedcentral.com/1471-2334/13/358/prepub
